# Combination Treatment of Persistent SARS-CoV-2 Infection with Dual Antiviral Therapy and Intravenous Immunoglobulin: A Novel Approach

**DOI:** 10.3390/jcm14248831

**Published:** 2025-12-13

**Authors:** Myrto Blizou, Stefanos Lampadakis, Emmanouil Karofylakis, Andromachi Blizou, Konstantinos Thomas, Spyridon Prountzos, Vasileios Papavasileiou, Thomas Raptakis, Effrosyni D. Manali, Spyros A. Papiris, Stelios Loukides, Elvira-Markela Antonogiannaki

**Affiliations:** 1 2nd Pulmonary Medicine Department, General University Hospital “Attikon”, Medical School, National and Kapodistrian University of Athens, 12462 Athens, Greece; myrto_bl@hotmail.com (M.B.); steflamp17@gmail.com (S.L.); vasilis1995pap@gmail.com (V.P.); tomraptakis76@yahoo.gr (T.R.); papiris@otenet.gr (S.A.P.); kantonogiannaki@gmail.com (E.-M.A.); 2 4th Department of Internal Medicine, General University Hospital “Attikon”, Medical School, National and Kapodistrian University of Athens, 12462 Athens, Greece; manoskarofilakis@gmail.com (E.K.); mahiblizou@hotmail.com (A.B.); costas_thomas@yahoo.com (K.T.); 3 2nd Department of Radiology, General University Hospital “Attikon”, Medical School, National and Kapodistrian University of Athens, 12462 Athens, Greece; spyttt@gmail.com

**Keywords:** persistent COVID-19, immunodeficiency, antivirals, immunoglobulin

## Abstract

**Background**: Immunocompromised patients, particularly those with humoral immune deficiencies or receiving B-cell-targeted therapies, are at increased risk of persistent SARS-CoV-2 infection, a condition often underrecognized and lacking standardized treatment. **Methods**: We present a case series of patients with persistent SARS-CoV-2 infection and underlying humoral immunodeficiency, treated at the General University Hospital “Attikon” from February 2023 to September 2024. Persistent infection was defined by prolonged symptoms, compatible imaging findings, and RT-PCR positivity beyond 21 days. All patients received combination antiviral therapy with remdesivir and nirmatrelvir/ritonavir, and intravenous immunoglobulin (IVIG), using a structured diagnostic and therapeutic algorithm. **Results**: Eleven patients (55% male), median age 56 [IQR 50–66] years, were included. Seven (64%) had hematologic malignancy, 10 (91%) received anti-CD20 therapy, and 6 (55%) had both. Median symptom duration before diagnosis was 63 [58–135] days. Ten (91%) experienced recurrent symptoms; one (9%) had progressive symptoms with severe respiratory failure requiring high-flow nasal cannula. Persistent infection was confirmed via bronchoscopy with bronchoalveolar lavage in 6 patients (55%). Prior to diagnosis, 5 patients (45%) required one hospitalization, 1 (9%) was hospitalized twice, and 2 (18%) had more than two hospitalizations. Following combination therapy, 10 (91%) achieved complete response at 180-day follow-up. **Conclusions**: The proposed diagnostic and therapeutic algorithm combining remdesivir, nirmatrelvir/ritonavir, and IVIG enhanced diagnostic value and therapeutic outcomes in this high-risk population.

## 1. Introduction

The novel coronavirus, SARS-CoV-2 was first identified in China in December 2019 and rapidly spread worldwide. To date, more than 770 million confirmed COVID-19 cases and over 7 million reported deaths have been documented [1]. The widespread implementation of vaccination, early administration of antiviral therapy to high-risk individuals, development of immunity through natural infection, and the emergence of new viral variants have collectively contributed to a decline in severe COVID-19 cases [2].

However, immunocompromised patients remain at increased risk of severe disease, often experiencing prolonged or recurrent viral shedding and persistent symptoms lasting for months, due to an inadequate immune response to infection or vaccination [3]. A systematic review indicated that viable viral shedding may persist for up to four months in immunocompromised individuals, with a median duration of 20 days. In contrast, among the general population, 95% of samples are no longer viable after day 15, with a median shedding duration of 11 days [4].

Patients with B-cell depleting diseases or those receiving B-cell-targeted therapies, particularly anti-CD20 treatment, are at increased risk of developing persistent COVID-19 infection [5]. This condition is marked by lingering or recurrent symptoms lasting over 7–14 days, along with compatible radiological findings and prolonged viral presence, even at low levels [5]. SARS-CoV-2 real-time reverse transcriptase-polymerase chain reaction (RT-PCR) tests remain persistently or intermittently positive beyond 21 days [6]. Symptom duration varies from several weeks to months, with cases reported lasting up to 300 days [6]. A recent publication proposed classifying persistent SARS-CoV-2 infection into three categories based on clinical presentation: asymptomatic or pauci-symptomatic, relapsing, and chronic [7].

The diagnosis of persistent SARS-CoV-2 infection remains challenging, as ongoing viral replication may facilitate the emergence of novel variants [7]. Despite growing knowledge of COVID-19, optimal strategies for its prevention, diagnosis, and management are still not well defined.

In this case series, we describe eleven patients with persistent SARS-CoV-2 infection and humoral immunodeficiency, and propose a novel diagnostic and therapeutic algorithm for managing these patients. The treatment approach combines antiviral drugs (remdesivir and nirmatrelvir/ritonavir) along with intravenous immunoglobulin (IVIG).

## 2. Materials and Methods

### 2.1. Study Population

**Figure 1:**
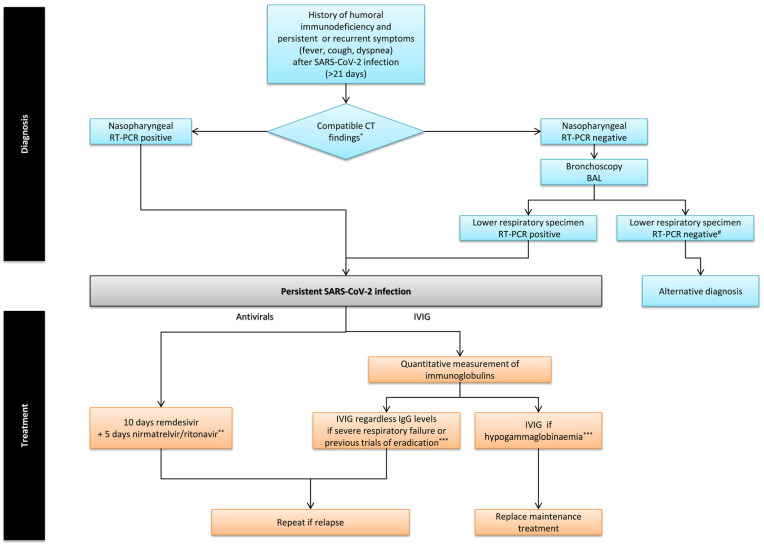
A diagnostic and therapeutic algorithm for patients with history of humoral immunodeficiency and persistent SARS-CoV-2 infection. CT = computed tomography scan of the chest, RT-PCR = real time reverse transcriptase-polymerase chain reaction, BAL = bronchoalveolar lavage, IVIG = intravenous immunoglobulin. * Compatible CT chest findings are bilatelal GGOs opacities and/or consolidations. ^#^ Repeat bronchoscopy if there is a strong clinical suspicion for persistent SARS-CoV-2 infection. ** Irrespective of previous administration. *** IVIG at a dose of 0.4–0.6 g/kg is recommended.

We describe a case series of patients managed between February 2023 and September 2024 at the General University Hospital “Attikon”in Athens, Greece. All patients were diagnosed with persistent SARS-CoV-2 infection and had an underlying humoral immunodeficiency. The diagnostic criteria for persistent SARS-CoV-2 infection are detailed below (Figure 1). Written informed consent was obtained from each participant. The study was approved by the Institutional Ethics Committee of the General University Hospital “Attikon” in Athens, Greece (ID: 487/3-9-2020).

### 2.2. Data Collection and Definitions

Collected data included demographic characteristics (age and sex), underlying hematologic comorbidities or other conditions requiring B-cell-targeted therapies, clinical presentation and symptoms, respiratory support requirements, and any previous anti-SARS-CoV-2 treatments. During the initial assessment, all patients underwent high-resolution computed tomography (HRCT) of the chest, and serum immunoglobulin levels were systematically measured.

### 2.3. Diagnostic Criteria for Persistent SARS-CoV-2 Infection

Patients were classified as having persistent SARS-CoV-2 infection if they met the following criteria:

1. Presence of humoral immunodeficiency (B-cell malignancy and/or ongoing or prior B-cell-targeted therapy);
2. Persistent or recurrent symptoms lasting longer than 21 days following the initial positive SARS-CoV-2 RT-PCR test;
3. Imaging findings consistent with COVID-19 infection, such as bilateral ground-glass opacities and/or pulmonary consolidations;
4. Persistent or intermittent SARS-CoV-2 RT-PCR positivity for more than 21 days.

For patients who fulfilled these criteria but had a negative nasopharyngeal RT-PCR result, further diagnostic evaluation was performed using bronchoscopy with bronchoalveolar lavage (BAL) to confirm persistent infection or to identify alternative diagnoses.

### 2.4. Assessment of Disease Severity

The severity of SARS-CoV-2-related disease was classified according to the World Health Organization (WHO) criteria for COVID-19 severity [2].

### 2.5. Follow-Up

Patients underwent clinical and radiological evaluation at three and six months post-treatment, including chest HRCT. Complete response was defined as full resolution of symptoms, clinical signs, and CT abnormalities associated with persistent SARS-CoV-2 infection. If complete response was achieved at three months, six-month follow-up included only clinical assessment.

### 2.6. Treatment Protocol

Patients received combination antiviral therapy with remdesivir (200 mg on day one, followed by 100 mg daily for 10 days) and nirmatrelvir/ritonavir (300/100 mg twice daily for 5 days). Additionally, intravenous immunoglobulin (IVIG, 0.4–0.6 g/kg) was administered to patients with hypogammaglobulinemia, severe disease, or history of treatment failure (Figure 1).

### 2.7. Statistics

Continuous variables were expressed as median (25–75th interquartile range, IQR) and compared using Wilcoxon signed-rank test, where appropriate. Categorical variables expressed as absolute numbers and percentages. All data analyses were performed using IBM SPSS Statistics version 26.0 (IBM Corp., Armonk, NY, USA).

## 3. Results

Eleven patients with persistent SARS-CoV-2 infection and underlying humoral immunodeficiency were included in this case series. The epidemiological, clinical and radiological characteristics of the patients are summarized in Table 1. The cohort consisted of 55% male patients, with a median age of 56 years (interquartile range [IQR]: 50–66 years). Among them, 7 patients (64%) had an underlying hematologic malignancy, including 2 with chronic lymphocytic leukemia (CLL) and 5 with non-Hodgkin lymphoma (NHL). A total of 10 patients (91%) had received anti-CD20 therapy within the last 8 months, with rituximab being the most commonly used agent (n = 8). Six patients (55%) had both hematologic malignancy and prior anti-CD20 treatment. Three patients (27%) received anti-CD20 therapy for autoimmune disorders, as indicated in Table 1. All patients have a history of vaccination with anti-SARS-CoV-2 mRNA vaccines, 5 with three doses, 4 with two doses and 2 with four doses (Table 1).

The median duration of symptoms before diagnosis was 63 days (IQR: 58–135 days). Persistent infection was confirmed via bronchoscopy with bronchoalveolar lavage (BAL) in 6 patients (55%). The median SARS-CoV-2 RT-PCR cycle threshold (CT) value obtained immediately before treatment initiation was 27 (IQR, 25.5–29). Hypogammaglobulinemia was identified in 7 patients (64%).

Recurrent symptoms were reported in 10 patients (91%), while 1 patient (9%) experienced progressive symptoms leading to severe respiratory failure requiring high-flow nasal cannula (HFNC) support. Among the treated patients, 10 had moderate disease, of whom 5 required low-flow oxygen support and one had severe disease requiring high-flow nasal cannula oxygen support.

Prior to establishing a diagnosis, 5 patients (45%) required one hospitalization, 1 (9%) patient was hospitalized twice and 2 (18%) had more than two hospitalizations. Before receiving the combination therapy, multiple eradication attempts were made: 4 patients received one prior treatment regimen consisting of a single antiviral agent with or without immunomodulators, 2 patients had received two regimens, 1 patient had undergone three, and 2 patients had received four or more treatment attempts (Table 1).

All patients received combination of antiviral drugs (remdesivir and nirmatrelvir/ritonavir) along with intravenous immunoglobulin (IVIG) (Table 1). All study patients met the safety criteria required for nirmatrelvir/ritonavir administration, including assessment of renal and hepatic function, as well as evaluation of potential drug–drug interactions. Hepatic and renal function tests were closely monitored during treatment, and no clinically significant abnormalities were observed. None of the patients received corticosteroids or immunomodulatory therapy, in accordance with the proposed algorithm (Figure 1).

Patients with hypogammaglobulinemia received maintenance immunoglobulin replacement therapy, as indicated in the proposed algorithm (Figure 1). The median serum IgG levels increased significantly from 3.5 g/L (IQR, 3.8–4.8) at baseline to 7.4 g/L (IQR, 8.5–12.1) after 3 months of substitution therapy (*p* = 0.028).

Following combination therapy, 10 patients (91%) achieved a complete response at 3 months and remained sustained during the 6-month follow-up. One patient (9%) with CLL, who had undergone multiple hospitalizations, remained infected for 384 days after the first positive SARS-CoV-2 RT-PCR test, and succumbed to fulminant *Clostridioides difficile* infection.

## 4. Discussion

Our preliminary clinical experience with combined antiviral therapy (remdesivir and nirmatrelvir/ritonavir) along with IVIG suggests potential effectiveness in the management of patients with humoral immunodeficiency and persistent SARS-CoV-2 infection. Complete resolution of symptoms and radiological abnormalities was observed in all patients except one, who experienced prolonged infection and finally died from other causes.

We propose that patients with humoral immune disorders who present with persistent or recurrent symptoms lasting more than 21 days after an initial positive SARS-CoV-2 test, compatible imaging findings (bilateral ground-glass opacities and/or consolidations), and persistent or intermittent positive SARS-CoV-2 RT-PCR beyond 21 days should be evaluated for persistent infection. In those with negative nasopharyngeal RT-PCR results, further diagnostic assessment via bronchoscopy is recommended to confirm persistent infection or to identify an alternative diagnosis (Figure 1).

Managing immunocompromised patients during the acute phase of COVID-19 remains challenging due to limited evidence and therapeutic uncertainty. Their exclusion from most clinical trials has resulted in a lack of robust data to guide management, leading to frequent relapses after standard therapy and increased morbidity and mortality. Remdesivir is recommended for both hospitalized and non-hospitalized patients with mild-to-moderate COVID-19 at risk of progression to severe disease [2]. However, immunocompromised individuals were largely underrepresented in these studies. A large retrospective analysis demonstrated that early initiation of remdesivir in hospitalized immunocompromised patients, including those not requiring supplemental oxygen, was associated with reduced mortality across variant waves [9]. NIH guidelines also recognize the risk of prolonged viral replication in this population and support extending remdesivir therapy when clinically indicated [2].

The EPIC-HR trial showed an 89% reduction in progression to severe disease with a 5-day course of oral nirmatrelvir/ritonavir in high-risk outpatients [2]. Although immunocompromised patients were underrepresented in this trial, subsequent retrospective studies have suggested some benefit in this population [10]. Two recent phase II trials (NCT05438602, NCT05567952) evaluated extended or repeated courses of nirmatrelvir/ritonavir in immunocompromised individuals. While the drug is authorized for non-hospitalized patients, one study in hospitalized immunocompromised patients showed shortened time to viral clearance with its use [11].

Data on the management of patients with persistent SARS-CoV-2 infection are even more limited. Case reports and small case series have suggested several approaches, including extended or repeated courses of remdesivir or nirmatrelvir/ritonavir, dual antiviral therapy combining both agents, and combination regimens involving antivirals with monoclonal antibodies or high-titer convalescent plasma [12,13,14,15].

Based on our proposed therapeutic algorithm, dual antiviral therapy is recommended for patients with persistent SARS-CoV-2 infection (Figure 1). Remdesivir monotherapy is often associated with treatment failure [3], and prolonged courses may increase the risk of viral resistance [16]. A recent study directly comparing dual antiviral therapy with conventional monotherapy in severely immunocompromised patients with persistent or recurrent SARS-CoV-2 infection demonstrated the superiority of the combination regimen, as monotherapy achieved only 50% effectiveness [17]. Concurrent use of remdesivir and nirmatrelvir/ritonavir appears to be well tolerated [13,15,17]. The rationale for dual therapy is the synergistic antiviral effect of agents with different mechanisms of action and the potential to prevent resistance. In our protocol, remdesivir is administered for 10 days, while nirmatrelvir/ritonavir is given for 5 days to minimize potential drug interactions.

Assessment of immunoglobulin levels is critical in patients with B-cell depleting conditions or therapies, given their increased risk for clinically significant hypogammaglobulinemia. Notably, in the context of anti-CD20 therapy, low immunoglobulin levels may persist long-term, even beyond 12 months after the last administration of the agent [18]. Immunoglobulin replacement is an established intervention for patients who develop clinically significant secondary hypogammaglobulinemia, often presenting with recurrent or persistent infections. Identifying such patients is essential, as they may benefit from IVIG for the prevention or treatment of infectious complications, including persistent SARS-CoV-2 infection, as outlined in our algorithm (Figure 1).

Currently, evidence supporting the use of IVIG for either acute or persistent COVID-19 in the absence of documented hypogammaglobulinemia remains limited. Nevertheless, recent studies have demonstrated that commercially available immunoglobulin preparations now contain substantial titers of neutralizing antibodies, reflecting widespread SARS-CoV-2 exposure and/or vaccination among plasma donors [19]. Although the clinical utility of long-acting monoclonal neutralizing antibodies has diminished due to reduced activity against Omicron subvariants [2], circulating preparations offer polyclonal antibodies with broader mechanisms of action, maintaining efficacy against emerging variants. Consequently, IVIG may represent a valuable adjunctive therapeutic option for patients with humoral immunodeficiency and persistent or relapsing COVID-19, even in the absence of confirmed hypogammaglobulinemia, particularly in cases of severe respiratory failure or after unsuccessful viral eradication attempts (Figure 1). Furthermore, a recently published consensus document by the European Society of Clinical Microbiology and Infectious Diseases (ESCMID) on the management of immunocompromised patients with COVID-19 highlights the potential role of IVIG in the treatment of severe persistent SARS-CoV-2 infection [20].

It is important to emphasize that our proposed algorithm advises against corticosteroids or other immunomodulatory therapies in these patients. Although the RECOVERY trial demonstrated the benefit of dexamethasone in COVID-19 patients with respiratory failure [3], its effect on mortality depends on disease severity, degree of inflammation, advanced age, and pre-existing immunosuppression. NIH guidelines recommend avoiding dexamethasone in immunosuppressed patients with minimal oxygen requirements early in the course of infection to prevent prolonged viral shedding [2]. Administering immunosuppressive therapy in cases of persistent infection may further impair viral clearance, promote viral dissemination, facilitate the emergence of new SARS-CoV-2 variants, and increase the risk of secondary bacterial infections.

The treated patient who eventually died from fulminant *Clostridioides difficile* infection had experienced prolonged SARS-CoV-2 persistence with recurrent symptoms and positive RT-PCR results for more than one year (384 days), despite multiple hospitalizations and treatments at other centers (Table 1). The repeated courses of corticosteroids, multiple hospitalizations, and extensive antimicrobial exposure likely acted as predisposing factors for the development of *Clostridioides difficile* infection. His clinical course further underscores the importance of early recognition and timely management of persistent SARS-CoV-2 infection in immunocompromised patients to optimize treatment.

Compared to previous publications [12,13,14,15,17], the present study proposes a diagnostic and therapeutic algorithm, emphasizing the need for early recognition and prompt bronchoscopy with BAL in patients with negative nasopharyngeal PCR but high clinical suspicion of persistent SARS-CoV-2 infection, as well as the assessment of immunoglobulin levels in patients with underlying humoral immunodeficiency and persistent infection. The safety and efficacy of dual antiviral therapy were confirmed in this study, consistent with prior reports [19]. Additionally, the value of IVIG is highlighted not only for patients with hypogammaglobulinemia but also due to the high titers of neutralizing antibodies present in commercial immunoglobulin products [20], which may enhance viral clearance in these patients.

Among the limitations of our study are its single-center, observational case series design, the small sample size, and the absence of a comparator group, all of which limit the generalizability of our findings. Furthermore, some laboratory tests, including confirmatory testing to assess virological clearance, were not performed, restricting the ability to draw definitive scientific conclusions.

In conclusion, vigilance is critical for the timely diagnosis and management of persistent SARS-CoV-2 infection in immunocompromised patients. Dual antiviral therapy with remdesivir and nirmatrelvir/ritonavir, combined with IVIG, appears to be a promising therapeutic approach. However, randomized controlled trials are needed to establish optimal diagnostic and therapeutic strategies for this vulnerable patient population.

## Figures and Tables

**Table 1:** The epidemiological and clinical characteristics of treated patients with persistent SARS-CoV-2 infection.

Case	Age/ Sex	Disease	Anti-CD20 Agent/ Last Dose	Vaccination History (Doses)	Days After 1st Positive SARS- CoV-2 Test	Previous SARS-CoV-2 Eradication Therapies (Number of Courses) *	Ct- RT- PCR	Symptoms	CT Findings (Extent, %) **	Respiratory Failure/ Support	IgG Levels (g/L)	Therapeutic Regimen	Outcome/ Response ***	Follow-Up (Days)
1	56/M	FCL	Rituximab/8 months	Yes (3)	120	RDV/CS (3) RDV/CS/Tocilizumab (1)	22	Fever/Dyspnea	GGOs/ Consolidations (10–25%)	No	Low (6.4)	RDV 10 days/NMV/r 5 days/ IVIG	Alive/ Complete	180
2	56/M	DLBCL	Obinutuzumab/2 months	Yes (2)	63	NMV/r (1) RD (2)	25	Fever	GGOs (10–25%)	No	Normal (11.6)	RDV 10 days/NMV/r 5 days/ IVIG	Alive/ Complete	180
3	78/M	RA	Rituximab/ 6 months	Yes (4)	44	No	29	Fever/Dyspnea/Cough	GGOs/ Consolidations (25–50%)	Yes/HFNC	Normal (9.1)	RDV 10 days/NMV/r 5 days/ IVIG	Alive/ Complete	180
4	84/M	CLL	No	Yes (4)	384	RDV/CS (4) RD (1)	26	Fever/Dyspnea/Cough	GGOs (10–25%)	Yes/low flow oxygen therapy	Low (2.6)	RDV 10 days/NMV/r 5 days/ IVIG	Deceased	-
5	24/F	GPA	Rituximab/ 4 months	Yes (3)	48	NMV/r (1) RDV/CS (1)	27	Fever/Cough	GGOs (10–25%)	No	Low (3.6)	RDV 10 days/NMV/r 5 days/ IVIG	Alive/ Complete	180
6	48/M	NLPHL	Rituximab/1 month	Yes (3)	60	No	27	Fever/Cough	GGOs (10–25%)	No	Low (3.7)	RDV 10 days/NMV/r 5 days/ IVIG	Alive/ Complete	180
7	69/F	CLL	Rituximab/ 6 months	Yes (3)	150	RDV/CS/ Tocilicumab (1)	29	Dyspnea	GGOs (25–50%)	Yes/low flow oxygen therapy	Low (4.3)	RDV 10 days/NMV/r 5 days/ IVIG	Alive/ Complete	180
8	63/F	NLPHL	Rituximab/ 8 months	Yes (2)	80	RDV/CS (1)	26	Fever	GGOs (10–25%)	No	Low (2.8)	RDV 10 days/NMV/r 5 days/ IVIG	Alive/ Complete	180
9	52/F	RA	Rituximab/ 8 months	Yes (2)	180	NMV/r (1) RDV (1)	25	Fever	GGOs (10–25%)	No	Low (3.9)	RDV 10 days/NMV/r 5 days/ IVIG	Alive/ Complete	180
10	38/F	MS	Ocrelizumab/ 6 months	Yes (2)	60	NMV/r (1)	27	Fever	GGOs (10–25%)	No	Normal (7.1)	RDV 10 days/NMV/r 5 days/ IVIG	Alive/ Complete	180
11	63/M	MCL	Rituximab/ 2 months	Yes (3)	55	RDV (1)	29	Fever/Cough	GGOs (10–25%)	No	Normal (7.2)	RDV 10 days/NMV/r 5 days/ IVIG	Alive/ Complete	180

* Previous eradication therapies were performed during prior hospitalizations or evaluations at other centers. ** Extent of COVID-19-associated pulmonary opacities [8]. *** As complete response is defined the complete resolution of symptoms, clinical signs and CT findings due to persistent SARS-CoV-2 infection. CT = computed tomography scan of the chest, Ct-RT-PCR = cycle threshold in real-time reverse transcriptase-polymerase chain reaction, F = Female, FCL = follicular center lymphoma, DLBCL= diffuse large B-cell lymphoma, CS = corticosteroids, GPA = granulomatosis with polyangiitis, IgG = Immunoglobulin G, M = Male, MCL= mantle cell lymphoma, MS = multiple sclerosis, NLPHL = nodular lymphocyte predominant Hodgkin lymphoma, NMV/r = nirmatrelvir/ritonavir, RA = rheumatoid arthritis, RDV = Remdesivir, CLL = chronic lymphocytic leukemia, GGOs = ground glass opacities, HFNC= high flow nasal cannula therapy, IVIG = intravenous immunoglobulin.

## Data Availability

All data generated or analyzed during this study are included in this article. Further enquiries can be directed to the corresponding author.
